# Simple and effective serum biomarkers potential for predicting status epilepticus in anti-N-methyl-D-aspartate receptor encephalitis

**DOI:** 10.1186/s12883-021-02545-6

**Published:** 2022-01-14

**Authors:** Yingying Liu, Lili Ma, Xiaomeng Ma, Xueying Ma, Jing Li, Donghong Li, Xiuli Lin, Zhumin Su, Xiaohong Chen

**Affiliations:** Department of Neurology, Third Affiliated Hospital, Sun Yat-sen University, 600 Tianhe Road, Guangzhou, 510630 Guangdong China

**Keywords:** Anti-N-methyl-D-aspartate receptor encephalitis, Status epilepticus, Combined detection, Predictor

## Abstract

**Background:**

Patients with anti-N-methyl-D-aspartate receptor (NMDAR) encephalitis who also present with status epilepticus (SE) often have a poor prognosis. The aim of this study is to explore simple and effective predictors for anti-NMDAR encephalitis accompanied with SE.

**Methods:**

We retrospectively analyzed 65 anti-NMDAR encephalitis patients from January 2015 to December 2018 who admitted to the Third Affiliated Hospital of Sun Yat-sen University. Patients were divided into SE group and non-SE groups. Their pre-treatment data and 3-month follow-up data were retrospectively analyzed.

**Results:**

The results showed that compared with the non-SE group, the levels of serum uric acid (UA) and high-density lipoprotein cholesterol (HDL-C) in anti-NMDAR encephalitis patients with SE decreased significantly before treatment. Additionally, the levels of serum UA and HDL-C increased while the level of C-reactive protein (CRP) decreased 3 months after treatment in the SE group. Compared with the non-SE group, the SE patients had higher modified Rankin scale (mRS) scores before (mRS1) and after treatment (mRS2). Serum UA concentrations before treatment showed significantly negative correlations with mRS1 (*r* = − 0.407, *p* < 0.01) and mRS2 (*r* = − 0.458, *p* < 0.001), while the level of serum CRP before treatment had strong positive correlations with mRS1 (*r* = 0.304, *p* < 0.05) and mRS2 (*r* = 0.301, *p* < 0.05) in anti-NMDAR encephalitis patients. The receiver operating characteristic curve demonstrated that the combined detection of UA, HDL-C and CRP before treatment had a significantly higher value (the area under the curve = 0.848; 95% confidence interval [CI], 0.74–0.957; *p* < 0.001) to predict anti-NMDAR encephalitis accompanied with SE than that of single detection.

**Conclusions:**

Hence, the combined detection of serum UA, HDL-C and CRP before treatment may be simple and effective indicators for predicting SE in anti-NMDAR encephalitis, which may be helpful in early stages to remind clinicians to be alert to the emergence of SE.

## Background

Anti-N-methyl-D-aspartate receptor (NMDAR) encephalitis, recognized by Dalmau et al. in 2007, is a rare autoimmune disorder of central nervous system that is often misdiagnosed [1]. Clinical signs and symptoms of anti-NMDAR encephalitis encompass prodromal symptoms, such as fever, flu-like illness or headaches from days to weeks before the onset, followed by neuropsychiatric changes, including memory deficits, coma, dyskinesia, seizures, and autonomic and language dysfunction [2]. It has been reported that 25% of anti-NMDAR encephalitis patients also present with status epilepticus (SE) [2, 3]. Different from the SE caused by any other etiologies, the SE secondary to anti-NMDAR encephalitis tends to respond less favorably to the standard anti-seizure drugs, while monitoring as well as hospitalization is frequent [4]. SE may lead to neuronal injury and/or death and neuronal networks changes, depending on the type and duration of seizures [5]. The mortality of non-refractory SE is close to 10%, whereas the mortality of refractory and super-refractory SE rises to 30 and 50% respectively [6]. After the acute phase of anti-NMDAR encephalitis with SE, the continuation of anti-epilepsy drugs (AEDs) still could be considered [7]. Therefore, explorations in the potential diagnostic markers or therapies are meaningful for these patients in the clinical practice.

It has been reported that the levels of reactive oxygen species (ROS) and reactive nitrogen species (RNS) are tightly related to SE [8]. Seizures can give rise to the accumulation of free radicals and hyper-excitability, increases in extracellular glutamate concentration, and overstimulation of the Ca2+ signaling pathways in mitochondria. These can result in mitochondrial dysfunction, oxidative stress and neuronal dysfunction or death, while cell death itself can cause an epileptic propagation reaction [8]. ROS and RNS are generated in the pathophysiological processes above [8]. Uric acid (UA) is a powerful ROS/RNS scavenger in human biological fluids and may represent the most abundant endogenous antioxidant activities against oxidative damage [9]. UA has been reported to exert neuroprotective effects in many neurological diseases such as Alzheimer’s disease, Parkinson’s disease, and multiple sclerosis [10]. Similarly, high-density lipoprotein (HDL) has also been shown to exert the important antioxidant activities and prevent vascular inflammation [11, 12]. C-reactive protein (CRP) is a major, classical, acute-phase, reactant protein [13, 14]. In SE, the serum concentrations of acute-phase reactant proteins are significantly increased [15, 16]. Therefore, we hypothesized that serum UA, HDL-C and CRP may have the potential to predict SE in anti-NMDAR encephalitis patients.

Here, we retrospectively analyzed 65 anti-NMDAR encephalitis patients from January 2015 to December 2018 who admitted to the Third Affiliated Hospital of Sun Yat-sen University. Their pre-treatment data and three-month follow-up data, especially the association between the three serum biomarkers level and SE were retrospectively analyzed. Since it is essential for clinicians to be able recognize SE early through a simple and effective means and determine the most effective treatment strategy, our study may provide evidence for potential predictors of SE in anti-NMDAR encephalitis patients.

## Methods

### Study design and participants

Anti-NMDAR encephalitis was diagnosed according to the diagnostic criteria established in 2016. Patients were presented with specific neurological features (such as psychiatric symptoms, seizures, movement disorders, disturbances of memory, speech disorder, impaired consciousness and central hypoventilation), and were positive for NMDAR antibodies in the cerebrospinal fluid (CSF) [1–3, 17]. Epilepsy was diagnosed referring to the criteria of the International League against Epilepsy [18, 19]. Tonic-clonic SE exceeding 5 min, absence SE and focal SE lasting longer than 10 min [20]. Video EEG was performed for 48 h after admission. Continued recordings were performed if epileptiform discharges were observed, and recordings were stopped if no discharges were observed. When the patient had convulsions or/and changes of behavior, state of consciousness and sensation, continuous video EEG recordings were started again. SE was diagnosed according to clinical manifestations and video EEG. Inclusion criteria were as follows: (1) age ≥ 14 and ≤ 60 years; (2) newly diagnosed as anti-NMDAR encephalitis prior to immunotherapies; (3) patients with complete clinical data, such as regular blood tests, serological tests (liver and kidney function test, blood lipid test, serum UA test, and serum CRP test), an antibody of NMDAR in CSF test, an electroencephalography (EEG) scan, a brain magnetic resonance imaging (MRI) scan and an abdominal ultrasound or computed tomography (CT) scan. Patients were excluded from the study when: (1) they had a history of gout, high UA hematic disease, familial hypercholesterolemia, rheumatism, liver and kidney function decline, diabetes, hepatitis, or cirrhosis; (2) suffered from infection, such as pulmonary infection or urinary tract infection; (3) received medications before admission that could affect measurements, such as steroids, aspirin, pyrazinamide, furosemide, allopurinol, hypolipidemic and HDL enhancing drugs (such as statins and fibrates); (4) showed a positive result for other autoimmune encephalitis antibodies.

In order to control the selection bias, two experienced researchers strictly screened subjects in accordance with the inclusion and exclusion criteria. The researchers searched and screened the cases individually using the electronic patient database of the Third Affiliated Hospital of Sun Yat-sen University from January 2015 to December 2018. The consistent anti-NMDAR encephalitis patients were enrolled in this study according to the inclusion and exclusion criteria. A total of 65 patients diagnosed with anti-NMDAR encephalitis were enrolled, and then divided into an SE group and a non-SE group according to their manifestations. There were 21 patients in the SE group (16 convulsive SE and five non-convulsive SE), and 44 in the non-SE group. Their pre-treatment and three-month post-treatment data were retrospectively analyzed. All anti-NMDAR encephalitis patients were followed up 3 months after their first discharge, and relevant tests and examinations were performed. We collected a total of 47 healthy controls, whose age and sex were matched with the anti-NMDAR encephalitis patients.

The modified Rankin scale (mRS) was used to assess the neurological status of anti-NMDAR encephalitis patients [21]; a higher mRS reflects a worse neurological status. The mRS scores before treatment was defined as mRS1, and the mRS scores 3 months after treatment was regarded as mRS2. The improved mRS score was the change of mRS after treatment compared with the one before treatment. Here, treatment included first-line immunotherapies (steroids, intravenous immunoglobulin, plasmapheresis), second-line immunotherapies (rituximab, cyclophosphamide), long-term immunosuppression (e.g., mycophenolate mofetil, azathioprine) and tumor removal [22, 23]. All patients received the same standard treatment. During hospitalization, all patients started high-dose methylprednisolone (1.0 g/d for 3–⁠7 days) and intravenous immunoglobulin (0.4 g/kg/d for 3–5 days) therapies immediately after being diagnosed with anti-NMDAR encephalitis. Rituximab was added for some patients. There were 9 non-SE patients and 4 SE patients received second-line immunotherapies. At the same time, the teratoma was removed as soon as possible. After discharge from the hospital, all patients were given low-dose oral prednisone. For some patients, long-term immunosuppression was added. There were 8 non-SE patients and 5 SE patients received long-term immunosuppression.

### Biochemical assays

All patients underwent blood tests, a CSF test and a brain MRI study within 24 h, 3 days, and 1 week after admission, respectively. When anti-NMDAR encephalitis was suspected according to the specific neurological features on admission, a CSF tests for IgG antibodies against NMDAR was performed as soon as possible. Therefore, the period from admission to treatment is nearly equal for all patients. All EEG data were recorded according to the international 10–20 system using an EEG DigiTrack (ELMIKO, Poland) with 19 electrodes. Abdominal ultrasound or CT scan was conducted to assess the presence of teratoma. Serum albumin (ALB), total bilirubin (TBILI), UA, creatinine (Cr), total cholesterol (CHOL), low density lipoprotein cholesterol (LDL-C), HDL-C and CRP concentrations were measured by a direct enzymatic method, using a Clinical Analyzer 7180-ISE (Hitachi High-Technologies, Japan). CSF samples were collected from all patients and analyzed with cell based assay (CBA) by a trained specialist in a laboratory with experience reading these assays. IgG antibodies against NMDAR detected by indirect immunostaining using a commercially available kit (EUROIMMUN Medizinische Labordiagnostika, Lübeck, Germany) according to the manufacturer’s instructions.

### Ethics approval and consent to participate

All experimental protocols were approved by the ethics committee of the Third Affiliated Hospital of Sun Yat-sen University. We confirm that we have read the journal’s position on issues involved in ethical publication, and all methods in this study were carried out in a way that is consistent with those guidelines and regulations. Because the subjects could not be contacted, this study was exempted from informed consent and approved by the ethics committee of the Third Affiliated Hospital of Sun Yat-sen University.

### Statistical analysis

Statistical analyses were conducted by the Statistical Package for the Social Sciences (SPSS) 25.0 software (IBM, Chicago, IL, USA). Continuous variables were expressed as mean ± standard deviation or median, while categorical variables were expressed as a count (percentage). The comparison of continuous variables between the two groups was analyzed with the independent t-test, and categorical variables were analyzed by Pearson’s Chi-square test or Fisher’s exact test. For multiple comparisons, a one-way analysis of variance (ANVOA) was performed to determine the differences between groups. Correlation analysis was conducted with Spearman’s correlation. The receiver operating characteristic (ROC) curve was used to analyze serological markers to assess their ability to distinguish SE patients. An area under the curve (AUC) > 0.7 was considered as a good predictive value. A *p*-value of *p* < 0.05 was considered statistically significant.

## Results

### Baseline demographic and clinical characteristics of 65 participants

Among 65 participants diagnosed with anti-NMDAR encephalitis enrolled in this study, 44 were non-SE patients aged 29.45 ± 11.75 years (19 males and 25 females), and 21 were SE patients aged 26.48 ± 9.44 years (10 males and 11 females). All patients were tested for UA, HDL-C and CRP. The average serum UA level (normal range: 90–420 μmol/L) of the SE group was significantly lower than the non-SE group (*p* < 0.01). The average UA level was 222.68 ± 77.53 μmol/L in the SE group and 288.82 ± 91.32 μmol/L in the non-SE group. The mean serum HDL-C level (normal range: 0.78–2.00 mmol/L) of the SE group was significantly lower than the non-SE group (*p* < 0.001). The average HDL-C level was 0.94 ± 0.25 mmol/L in the SE group and 1.33 ± 0.56 μmol/L in the non-SE group. Compared with the non-SE group, patients with SE had lower serum UA (*p* < 0.01) and HDL-C (*p* < 0.001) levels. While the SE group presented with a higher CRP level (normal range: 0.0–6.0 mg/L) compared with the non-SE group (7.96 ± 8.95 mg/L versus 3.59 ± 4.03 mg/L), the difference was not statistically significant (*p* = 0.069). (Table 1).

**Table 1:** Clinical manifestations of anti-NMDAR encephalitis patients with and without SE

	non-SE (*n* = 44)	SE (*n* = 21)	*p*-Value
Age (years)	29.45 ± 11.75	26.48 ± 9.44	0.314
Gender (female) (n, %)	25(56.82%)	11(52.38%)	0.736
Symptom (n, %)			
Psychiatric symptoms	38(86.36%)	19(90.48%)	0.699
Speech disturbances	10(22.73%)	2(9.52%)	0.309
Memory deficits	23(52.27%)	6(28.57%)	0.072
Movement disorders	12(27.27%)	10(47.62%)	0.105
Loss of consciousness	17(38.64%)	11(52.38%)	0.295
Central hypoventilation	7(15.91%)	5(23.81%)	0.443
Time from first symptom to diagnosis (median, days)	30	15	0.026
ALB, g/L	40.21 ± 5.10	39.79 ± 6.79	0.785
TBILI, μmol/L	10.19 ± 4.73	12.36 ± 13.49	0.365
UA, μmol/L	288.82 ± 91.32	222.68 ± 77.53	0.008
Cr, μmol/L	61.99 ± 19.19	64.00 ± 20.73	0.702
CHOL, mmol/L	4.04 ± 0.97	4.14 ± 0.74	0.295
LDL-C, mmol/L	2.92 ± 0.92	2.75 ± 0.64	0.464
HDL-C, mmol/L	1.33 ± 0.56	0.94 ± 0.25	0.000
CRP, mg/L	3.59 ± 4.03	7.96 ± 8.95	0.069
Brain MRI abnormality	21(47.73%)	6(28.57%)	0.143
Teratoma presence	8(18.18%)	4(19.05%)	1.000
Brain MRI abnormality	21(47.73%)	6(28.57%)	0.143
Teratoma presence	8(18.18%)	4(19.05%)	1.000
Second-line immunotherapy	9(20.45%)	4(19.05%)	1.000
Long-term immunosuppression	8(18.18%)	5(23.81%)	0.596

Data were presented as mean ± standard deviation, median or count (percentage), comparison between the two groups was determined by *t* test or *chi-square* test. *p* < 0.05 was considered significant. Non-SE, non-status epilepticus; *SE* status epilepticus, *anti-NMDAR* anti-N-methyl-D-aspartate receptor, *ALB* albumin, *TBILI* total bilirubin, *UA* uric acid, *Cr* creatinine, *CHOL* total cholesterol, *LDL-C* low density lipoprotein cholesterol, *HDL-C* high density lipoprotein cholesterol, *CRP* C-reactive protein, *MRI* brain magnetic resonance imaging.

Data were presented as mean ± standard deviation, median or count (percentage), comparison between the two groups was determined by *t* test or *chi-square* test. *p* < 0.05 was considered significant. Non-SE, non-status epilepticus; SE, status epilepticus; anti-NMDAR, anti-N-methyl-D-aspartate receptor; ALB, albumin; TBILI, total bilirubin; UA, uric acid; Cr, creatinine; CHOL, total cholesterol; LDL-C, low density lipoprotein cholesterol; HDL-C, high density lipoprotein cholesterol; CRP, C-reactive protein; MRI, brain magnetic resonance imaging.

**Fig. 1:**
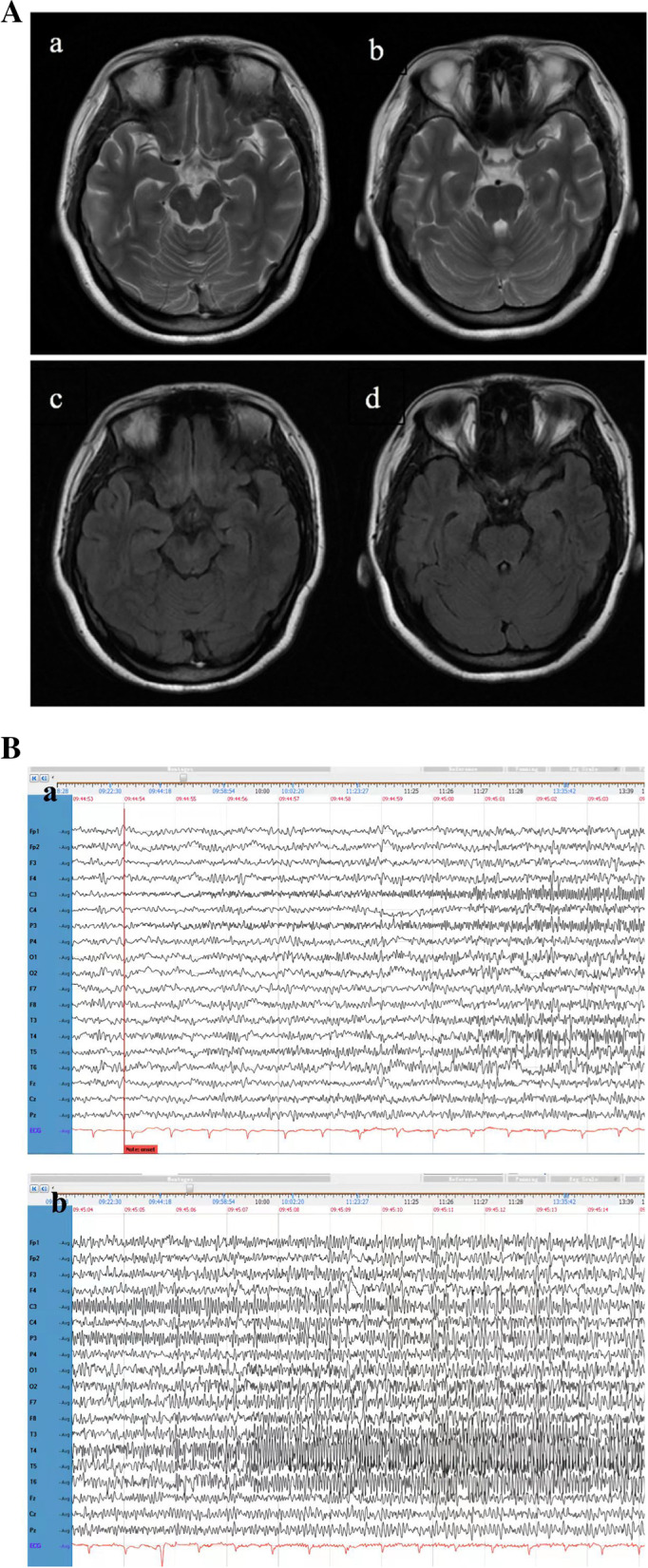
(A) Abnormal MRI signaling of one 28-year-old woman patient from the anti-NMDAR encephalitis with non-SE group. The brain MRI showed swelling and T2 FLAIR slightly hyperintensity in the bilateral temporal lobes. (a, b) T2-weighted MRI, (c, d) Axial T2 FLAIR sequences. MRI, magnetic resonance imaging; FLAIR, fluid attenuated inversion recovery; non-SE, non-status epilepticus; anti-NMDAR, anti-N-methyl-D-aspartate receptor. (B) A representative EEG of the anti-NMDAR encephalitis patients with SE. High frequency beta activity in the left central and parietal lobe with progressively increasing amplitude, typical of the tonic phase in status epilepticus, was associated in this patient with dystonic posturing of his right arm (a and b are continuous records). anti-NMDAR, anti-N-methyl-D-aspartate receptor; SE, status epilepticus

In brain MRI scans, 21 cases from the non-SE group and 6 cases from the SE group showed abnormalities. The most common abnormalities were in the temporal lobes. Other affected sites were the frontal lobes, basal ganglia, hippocampus, cerebellum, the insula and thalamus. As shown in Fig. 1A, a 28-year-old woman diagnosed with anti-NMDAR encephalitis without SE showed swelling and a slight T2-weighted-fluid-attenuated inversion recovery (FLAIR) hyperintensity in the bilateral temporal lobes.

All anti-NMDAR encephalitis with SE patients exhibited abnormalities in EEG. (Fig. 1B) The brain MRI of a 31-year-old man in the patient group was normal, but his EEG revealed a background of 10–12 Hz alpha activity, dense beta activity, and a sparse appearance of low voltage theta activity. Seizures consisted of a gradual increase in voltage of fast wave activity in the left central and parietal lobes for runs of 10 s. Subsequently, the generalized spike wave rhythm appeared, gradually evolved into a sharp wave rhythm, and finally returned to baseline levels. These bursts occurred every 4–5 min. The EEG patterns were accompanied with decreased consciousness, which is consistent with right upper limb tonic-clonic seizure. (Fig. 1B).

### The levels of serum UA and HDL-C decreased before treatment and increased 3 months after treatment in anti-NMDAR encephalitis patients with SE. The level of CRP in the SE group decreased 3 months after treatment

**Fig. 2:**
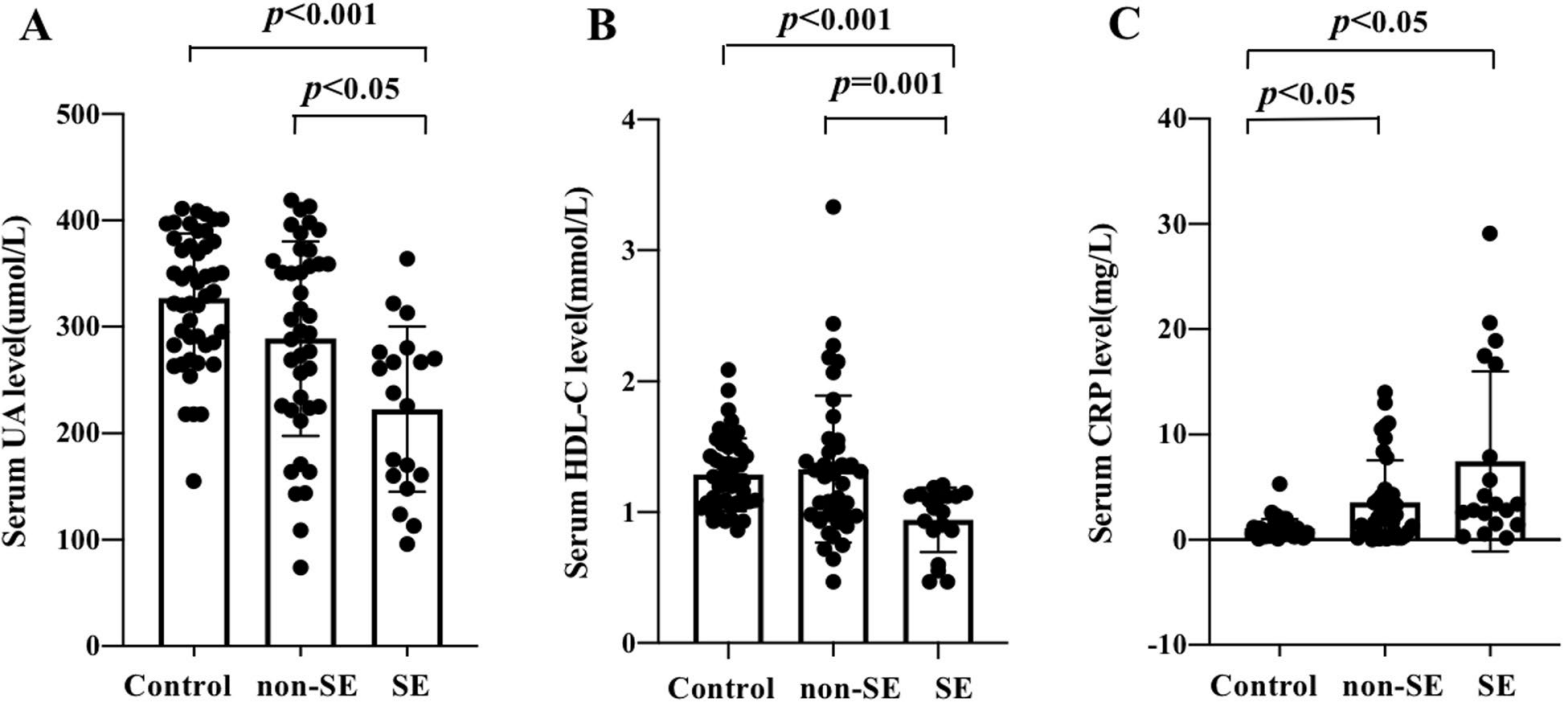
Comparison of serum UA, HDL-C and CRP before treatment among healthy controls, non-SE and SE anti-NMDAR encephalitis patients. *p* < 0.05 was considered significant. **A** uric acid; **B** high density lipoprotein cholesterol; **C** C-reactive protein. Non-SE, non-status epilepticus; SE, status epilepticus; anti-NMDAR, anti-N-methyl-D-aspartate receptor; UA, uric acid; HDL-C, high density lipoprotein cholesterol; CRP, C-reactive protein

The levels of serum UA, HDL-C and CRP before treatment in healthy controls, the non-SE group and the SE group are shown in Fig. 2. The serum UA level declined in the SE group compared with the healthy controls (*p* < 0.001) and the non-SE group (*p* < 0.05). There were no significant differences in serum UA levels between healthy controls and the non-SE group (*p* > 0.05). (Fig. 2A) Similarly, serum HDL-C levels fell in SE group compared with the healthy controls (*p* < 0.001) and the non-SE group (*p* < 0.01). There were no significant differences in serum HDL-C levels between healthy controls and the non-SE group (*p* > 0.05). (Fig. 2B) The serum CRP levels of the non-SE and SE groups were elevated significantly compared with the healthy controls (*p* < 0.05, *p* < 0.05). The level of serum CRP trended upward in the SE group compared with the non-SE group, although this difference was not statistically significant (*p* = 0.069). (Fig. 2C).

The patients were followed up for 3 months after treatment. In the non-SE group, there were no significant changes in UA, HDL-C and CRP serum levels 3 months after treatment (*p* > 0.05). In the SE group, UA (*p* < 0.01) and HDL-C (*p* < 0.05) serum levels increased, and CRP (*p* < 0.05) levels decreased significantly 3 months after treatment. There were no significant differences in UA, HDL-C and CRP serum levels between the two groups 3 months after treatment (*p* > 0.05). (Table 2).

**Table 2:** Serum UA, HDL-C and UA levels in the two groups of anti-NMDAR encephalitis patients

Variables	non-SE		SE		p1	p2	p3
	Before treatment	After treatment	Before treatment	After treatment			
UA	288.82 ± 91.32	292.18 ± 80.48	222.68 ± 77.53	310.75 ± 79.95	0.866	0.003	0.467
HDL-C	1.33 ± 0.56	1.33 ± 0.45	0.94 ± 0.25	1.17 ± 0.26	0.999	0.018	0.274
CRP	3.59 ± 4.03	3.10 ± 3.81	7.96 ± 8.95	1.61 ± 0.26	0.624	0.010	0.068

*p*1: before treatment vs. 3 months after treatment of no-SE group; *p*2: before treatment vs. 3 months after treatment of SE group; *p*3: non-SE group vs. SE group 3 months after treatment; anti-NMDAR, anti-N-methyl-D-aspartate receptor; non-SE, non-status epilepticus; *SE* Status epilepticus, *UA* Uric acid, *HDL-C* High density lipoprotein cholesterol, *CRP* C-reactive protein.

### Correlation between serum levels of UA, HDL-C, and CRP before treatment and mRS scores in anti-NMDAR encephalitis

**Fig. 3:**
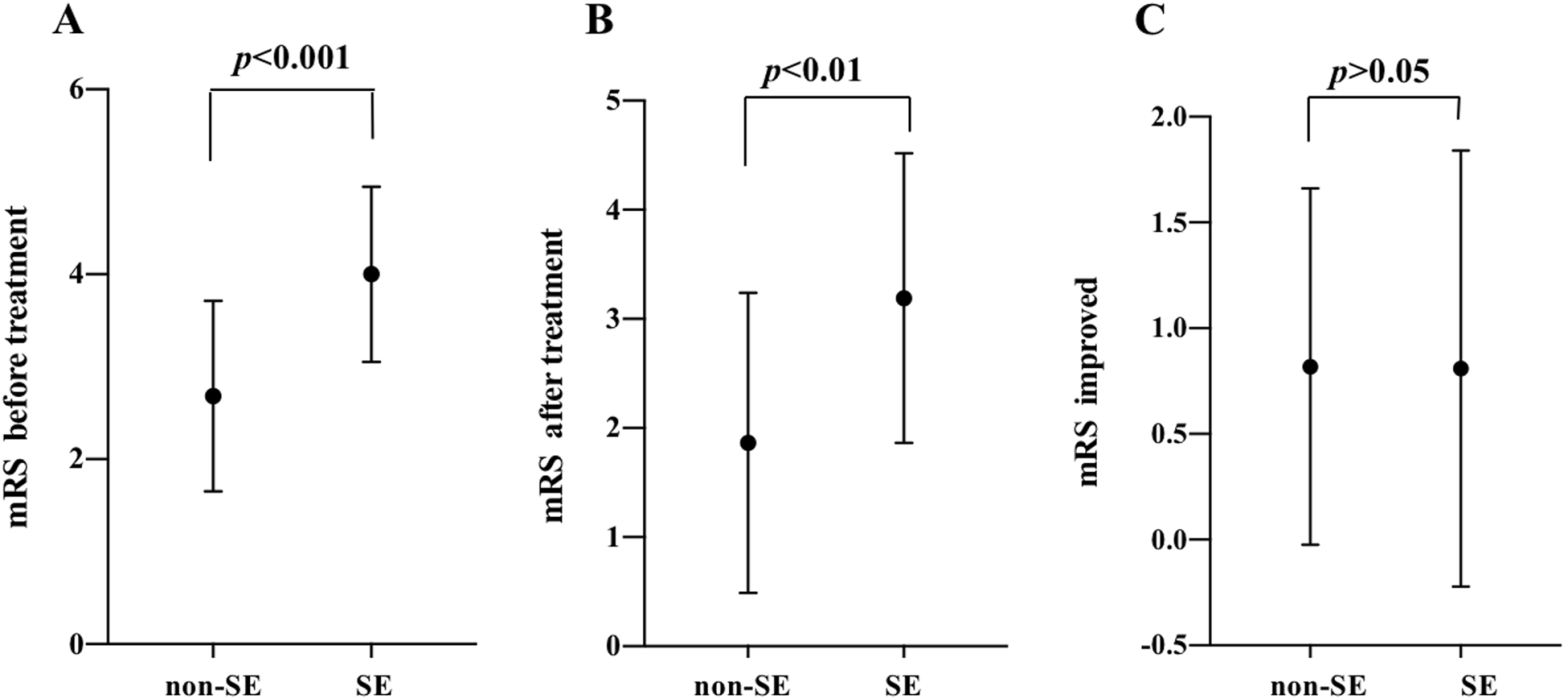
Comparison of mRS scores before treatment, after treatment and improved between non-SE and SE anti-NMDAR encephalitis patients. *p* < 0.05 was considered as significant. **A** mRS scores before treatment; **B** mRS scores after treatment; **C** mRS scores improved. The improved mRS scores was the change of mRS scores after treatment compared with before treatment. Non-SE, non-status epilepticus; SE, status epilepticus; anti-NMDAR, anti-N-methyl-D-aspartate receptor; mRS, modified Rankin scale

The mRS (before treatment, after treatment and improved) of anti-NMDAR encephalitis patients is shown in Fig. 3. Compared with the non-SE group, the mRS scores before treatment of the SE group was significantly increased (*p* < 0.001), (Fig. 3A) and the mRS scores after treatment of the SE group were higher than those of the non-SE group (*p* < 0.01). (Fig. 3B) However, the improved mRS scores of the non-SE group and SE group were nearly equal (*p* > 0.05). (Fig. 3C).

**Fig. 4:**
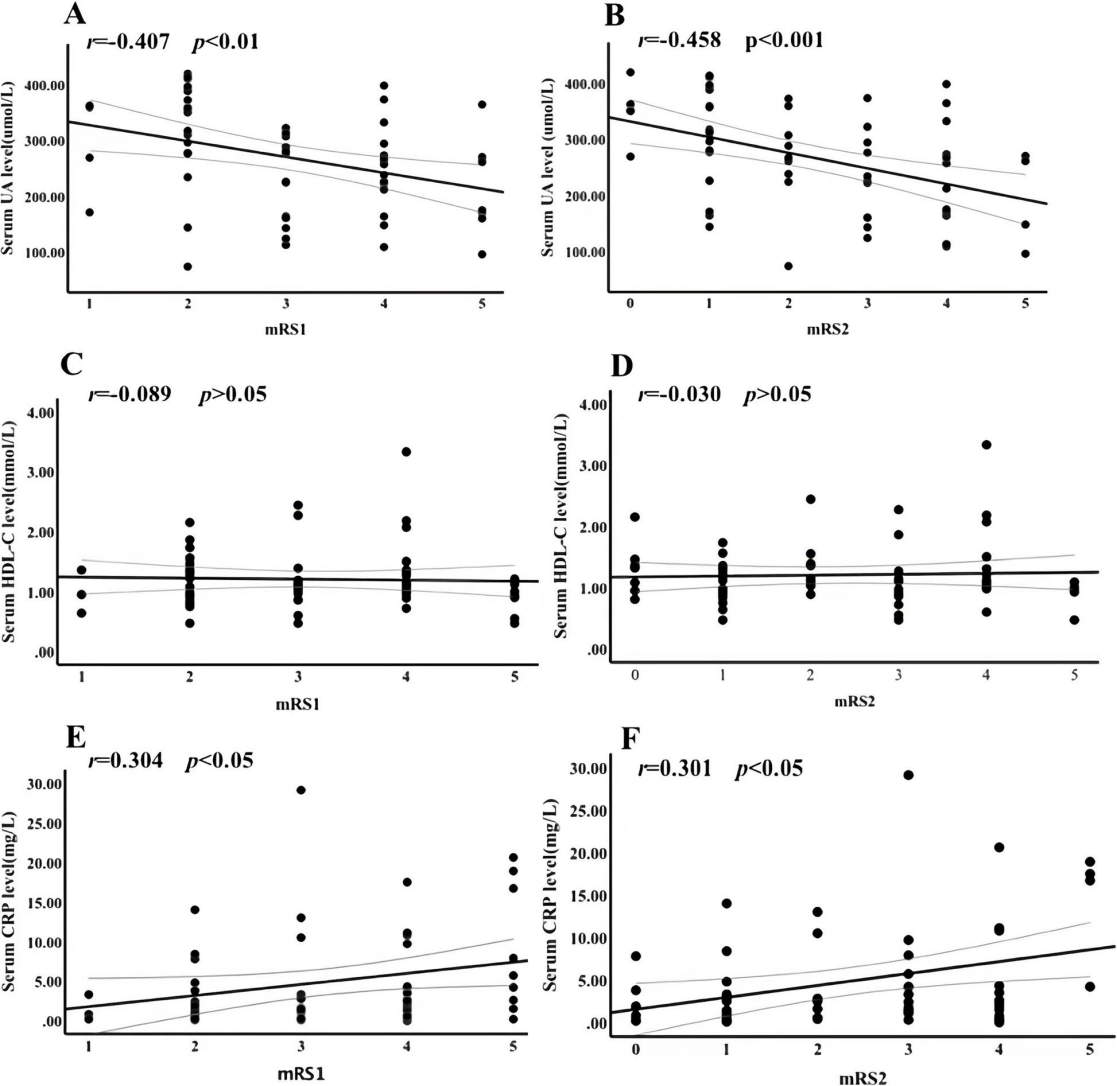
Correlation between serologic markers before treatment and mRS scores of anti-NMDAR encephalitis patients. *p* < 0.05 was considered significant. **A** negative correlation between UA and mRS scores before treatment (mRS1)(*r* = − 0.407, *p* < 0.01); **B** negative correlation between UA and mRS scores after treatment (mRS2) (*r* = − 0.458, *p* < 0.001); **C** correlation between HDL-C and mRS1(*r* = − 0.089, *p* > 0.05); **D** correlation between HDL-C and mRS2(*r* = − 0.030, *p* > 0.05); **E** positive correlation between CRP and mRS1(*r* = 0.304, *p* < 0.05); **F** positive correlation between CRP and mRS2(*r* = 0.301, *p* < 0.05). mRS, modified Rankin scale; mRS1, mRS scores before treatment; mRS2, mRS scores after treatment; anti-NMDAR, anti-N-methyl-D-aspartate receptor; UA, uric acid; HDL-C, high density lipoprotein cholesterol; CRP, C-reactive protein

In anti-NMDAR encephalitis, we found that there was a significant strong negative correlation between mRS1 and UA serum concentrations before treatment (*r* = − 0.407, *p* < 0.01). (Fig. 4A) Additionally, UA serum concentrations before treatment showed a strong negative correlation with mRS2 (*r* = − 0.458, *p* < 0.001). (Fig. 4B) There was no significant correlation between HDL-C serum levels before treatment and mRS1 (*r* = − 0.089, *p* > 0.05) (Fig. 4C) or mRS2 (*r* = − 0.030, *p* > 0.05). (Fig. 4D) CRP serum levels before treatment had a significantly positive correlation with mRS1 (*r* = 0.304, *p* < 0.05) (Fig. 4E) and mRS2 (*r* = 0.301, *p* < 0.05). (Fig. 4F).

### Combined detection of UA, HDL-C and CRP before treatment is a potential predictor of SE in anti-NMDAR encephalitis

**Fig. 5:**
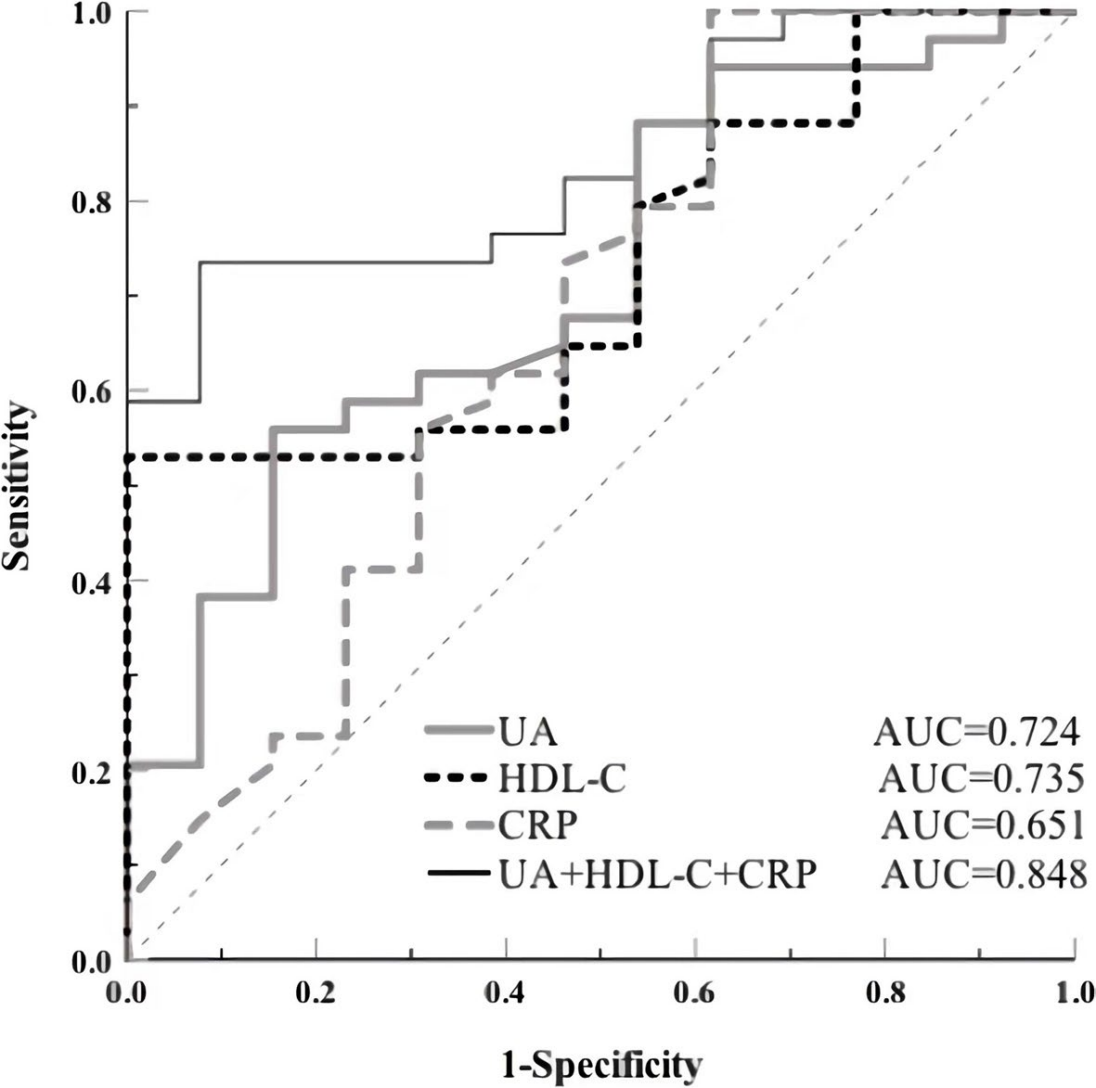
The receiver operating characteristic (ROC) analysis for models predicting SE in anti-NMDAR encephalitis. Combined UA, HDL-C and CRP levels before treatment exhibited the largest AUC (0.848) of the serologic parameters that were investigated, revealed had good value in distinguishing SE from non-SE. ROC, receiver operating characteristic; SE, status epilepticus; non-SE, non-status epilepticus; anti-NMDAR, anti-N-methyl-D-aspartate receptor; UA, uric acid; HDL-C, high density lipoprotein cholesterol; CRP, C-reactive protein; AUC, area under the ROC curve. ROC curve analysis

As shown in Fig. 5, the AUC of the best fitted model (AUC 0.848; 95% confidence interval [CI], 0.74–0.957; *p* < 0.001) was significantly higher than those of the univariate models, including indictors UA (AUC 0.724; 95% CI, 0.566–0.883; *p* < 0.05), HDL-C (AUC 0.735; 95% CI, 0.589–0.881; *p* < 0.05), and CRP (AUC 0.651; 95% CI, 0.489–0.813; *p* > 0.05).

## Discussion

This retrospective study demonstrated that the serum levels of UA, HDL-C and CRP before treatment has a potential to distinguish SE and non-SE in anti-NMDAR encephalitis patients and could help determine the severity and the prognosis of the disease. The ROC curve showed that the combined detection of UA, HDL-C and CPR serum levels before treatment had a significantly higher value in predicting SE than that of any single detection in anti-NMDAR encephalitis. Therefore, simultaneous detection of these three serum indicators may be a predictor of SE in anti-NMDAR encephalitis.

Anti-NMDAR encephalitis is an inflammatory disease mediated by anti-neuronal antibodies [ [24]]. Clinical observations and animal models have shown that both oxidative stress and neuro-inflammation are implicated in the pathogenesis of SE patients [25]. SE patients have significant changes in oxidative stress status parameters [26]. As the final product of purine metabolism, UA is an important low molecular weight antioxidant and powerful radical scavenger, which provides protection against oxidative stress in humans [25]. Our data demonstrate that the levels of serum UA before treatment are lower in anti-NMDAR encephalitis patients with SE when compared with the non-SE group and are higher 3 months after treatment. As an endogenous antioxidant, UA can reduce the release of proinflammatory cytokines and remove ROS [27]. In anti-NMDAR encephalitis patients, UA serum concentrations were much lower than in the healthy controls, especially in patients with more serious and poorer prognosis [28]. In the present study, the level of serum UA before treatment was related to the severity of the anti-NMDAR encephalitis. These results are in agreement with a previous report [28], which illustrated that the decrease of UA before treatment not only increases the risk of an SE attack, but also suggests more serious disease.

The decrease of HDL-C levels before treatment in anti-NMDAR encephalitis patients with SE may mirror what is seen with UA. Like UA, HDL particles have also been reported to have antioxidant and anti-inflammatory properties [29]. The main enzyme on their surfaces, paraoxonase 1 (PON1), plays an important role in the antioxidant properties of HDL-C [29]. Our results demonstrate that HDL-C serum levels also decrease in anti-NMDAR encephalitis patients with SE when compared with the non-SE group, and they increase 3 months after treatment. This indicates that HDL-C has a weaker antioxidant capacity in SE patients. PON1 activity has been observed to decrease significantly in the course of relapsing-remitting multiple sclerosis relapses [30]. However, serum lipid levels have modest effects on changes in multiple sclerosis disability, and HDL-C levels were not associated with expanded disability status scale worsening in MS. [31] Similarly, no significant correlation between HDL-C serum levels and the severity of the anti-NMDAR encephalitis was discovered in the present study. Here, we may explain it with the concept of “dysfunctional” HDL-C, which refers to HDL-C with pro-inflammatory properties [32]. Our results indicate that, even if serum HDL-C was involved in anti-NMDAR encephalitis with SE as an antioxidant, its role in the adaptation of disease seriousness is far from being established.

Since inflammation and immunity are considered core factors for anti-NMDAR encephalitis [ [24]], we next assessed the levels of serum CRP in anti-NMDAR encephalitis patients. CRP is a major, classical, acute-phase, reactant protein and a versatile component of human innate host defense mechanism that can also reflect the degree of an inflammatory response [33]. CRP levels from the anti-NMDAR encephalitis patients were significantly higher than healthy controls and significantly reduced after treatment [34]. In our study, both the non-SE and SE groups presented clearly higher CRP levels when compared with healthy controls, especially in the SE group. Additionally, the CRP levels of the SE group decreased 3 months after treatment. This suggests that the inflammatory response in anti-NMDAR encephalitis patients with SE may be more intense. Our results also suggest that the CRP serum levels are related to the severity of anti-NMDAR encephalitis both before and after treatment. Thus, elevated circulating levels of CRP may be associated with the severity and poor prognosis of anti-NMDAR encephalitis in patients and also an increased risk of SE.

In anti-NMDAR encephalitis patients, both UA and HDL-C serum levels were lower and CRP levels were higher than healthy controls, but all three significantly improved after treatment [28, 34, 35]. We performed a more in-depth subgroup analysis of patients with anti-NMDAR encephalitis. Our results suggest that the levels of UA, HDL-C, and CRP change significantly before and after treatment only in the SE group. We speculate that the more severe declined antioxidant capabilities and inflammation, more likely to suffer SE [25, 26]. The three biomarkers had improved after treatment, which may be associated with ameliorative tissue damage and inflammation of the central nervous system [27, 33]. The levels of the three biomarkers before treatment in non-SE group were close to those in healthy controls. It may indicate that the antioxidant capacity damaged not seriously and the inflammatory is slight in non-SE anti-NMDAR encephalitis patients. Nevertheless, further studies will be needed to elaborate the potential mechanisms.

There are still some limitations in this study. Firstly, since it is a retrospective study, further exploration into the associations between UA, HDL-C and CRP serum levels and the long-term outcome of SE in anti-NMDAR encephalitis patients are needed. Additionally, with being a single-center study with a short follow-up of a small sample size that was analyzed by only single variable analyses, also limits the interpretations of the study. Finally, although all patients received standard treatment, the potential effects of treatment remained a confounding variable.

This study innovatively found that the changes of serum UA, HDL-C and CRP levels in anti-NMDAR encephalitis patients with SE, and further explore the serum biomarkers correlation with mRS scores of anti-NMDAR encephalitis patients. Besides, the combined detection of UA, HDL-C and CRP had a significantly higher value to predict SE than that of any single detection in anti-NMDAR encephalitis. The creativity of this study is making use of three simple, convenient, routinely available serum biomarkers to predict SE in anti-NMDAR encephalitis, which may be helpful in early stages to remind clinicians to be alert to the emergence of SE. By detecting the levels of UA, HDL-C and CRP, the possibility of SE can be evaluated and acted upon quickly. Of course, this type of diagnosis of SE should be combined with clinical manifestations and EEG studies. In addition, this study also inspires the research of antioxidant therapies, especially additional supplements of UA and HDL-C in anti-NMDAR encephalitis patients with SE.

## Conclusions

This retrospective study suggests that the variation in the serum levels of UA, HDL-C and CRP before treatment may serve as significant biomarkers for anti-NMDAR encephalitis patients with SE. The combined detection of UA, LDL-C and CRP before treatment may be a predictor of SE in anti-NMDAR encephalitis patients.

## Data Availability

The datasets used and/or analyzed during the current study are available from the corresponding author on reasonable request.

## Abbreviations

anti-NMDAR: Anti-N-methyl-D-aspartate receptor
SE: Status epilepticus
non-SE: Non-status epilepticus
UA: Uric acid
HDL-C: High density lipoprotein cholesterol
CRP: C-reactive protein
ROC: Receiver operating characteristic
CSF: Cerebrospinal fluid
EEG: Electroencephalography
MRI: Brain magnetic resonance imaging
CT: Computed tomography
mRS: Modified Rankin scale
ALB: Albumin
TBILI: Total bilirubin
Cr: Creatinine
CHOL: Total cholesterol
LDL-C: Low density lipoprotein cholesterol
ROS: Reactive oxygen species
RNS: Reactive nitrogen species
AUC: Area under the curve
CBA: Cell based assay
PON1: Paraoxonase 1
EDSS: Expanded Disability Status Scale
